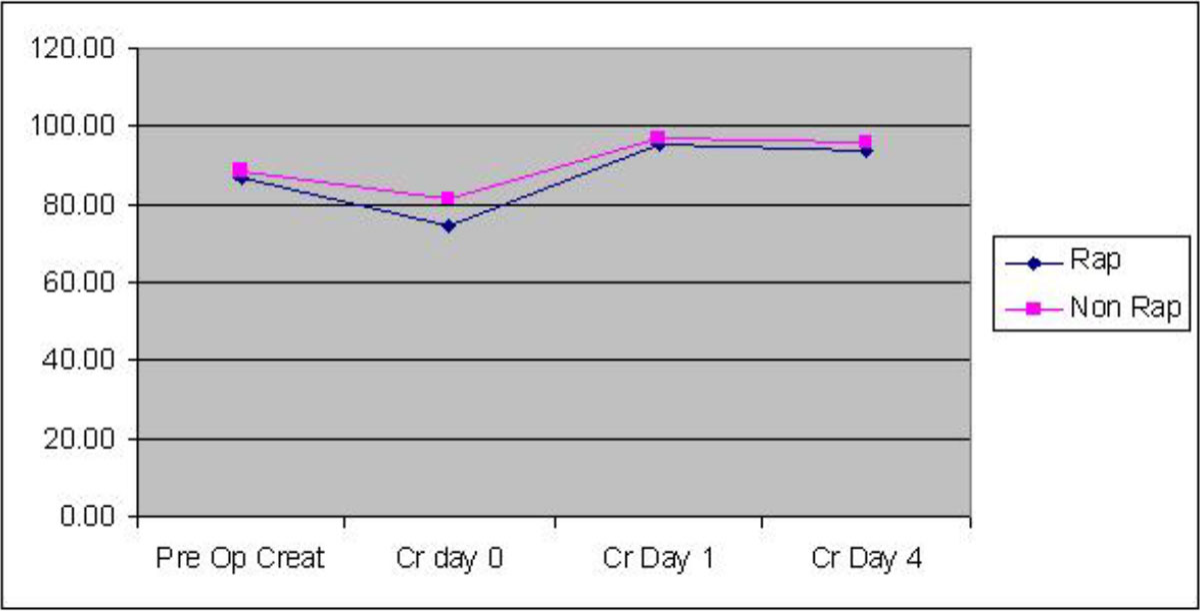# Does retrograde autologous priming of the cardiopulmonary bypass circuit have an impact on haematocrit and blood transfusion in uncomplicated coronary artery bypass graft surgery? A retrospective analysis

**DOI:** 10.1186/1749-8090-10-S1-A182

**Published:** 2015-12-16

**Authors:** Royan Richards, Rajani Rajnish, Aamir Khan, Hassan Zeinah, Katherine Farley, Diana Hughes, Yvonne Ashworth, Angela Wilby, Amal Bose, Antony Walker

**Affiliations:** 1 Department of Cardiothoracic Surgery, Lancashire Cardiac Centre- Blackpool Victoria Hospital, Whinney Heys Road, Blackpool, FY38NR, UK

## Background/Introduction

Haemodilution occuring as a result of cardiopulmonary bypass prime volume increases the need for transfusion of allogenic blood products. Many techniques have been employed to reduce the same. Priming the CPB circuit with the patient's own blood is thought to decrease haemodilution and transfusion requirements.

## Aims/Objectives

We studied the impact of Retrograde Autologous Priming(RAP) of the CPB circuit in patients undergoing uncomplicated coronary artery surgery to assess haematocrit levels and transfusion needs.

## Method

We did a retrospective study on two groups of patients undergoing coronary artery bypass surgery over a six month period. In the non RAP group (n = 124), the CPB circuit was primed with crystalloid standard prime. In the RAP group (n = 120), retrograde autologous priming was used to reduce crystalloid prime (620.77 ml ± 133.13 ml). Haematocrit levels, transfusion requirements and other clinical parameters were evaluated.

Data collection from various databases in the hospital was done using Microsoft excel spreadsheet. Statistical analysis was performed using SPSS software for windows.

## Results

Demographic data and operative parameters were equal for patients in both groups.

The haematocrit levels pre CPB, lowest Hct on CPB and end of CPB were 37.9, 26.4, 27.43 in the RAP group compared to 41.35, 28.00, 28.36 in the Non RAP group.

Blood transfusion requirements in the RAP group was 0.84 and 0.80 in the Non RAP group. There was no significant difference in length of stay, post op AF.

## Discussion/Conclusion

Our analysis concludes that RAP of the CPB circuit does not significantly increase haemotocrit levels or reduces blood transfusion requirements in patients having uncomplicated coronary artery bypass graft surgery. Haematocrit levels may have been even lower without RAP in the study group. Hence, further studies are required to assess RAP benefits in patients having coronary and valvular cardiac operations.

**Figure 1:**
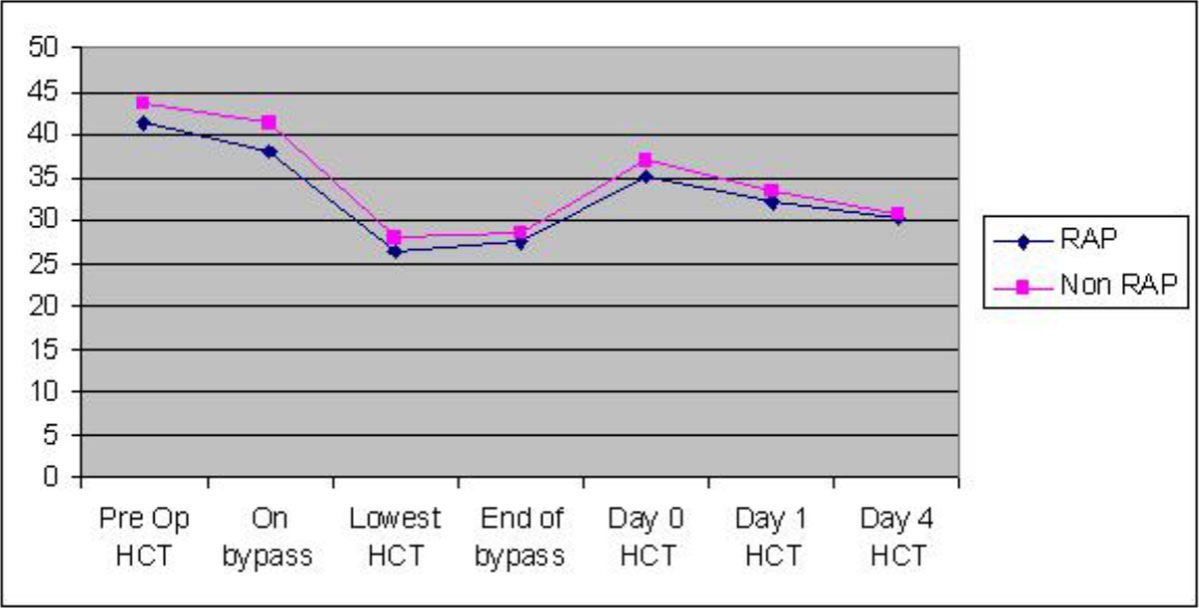


**Figure 2:**
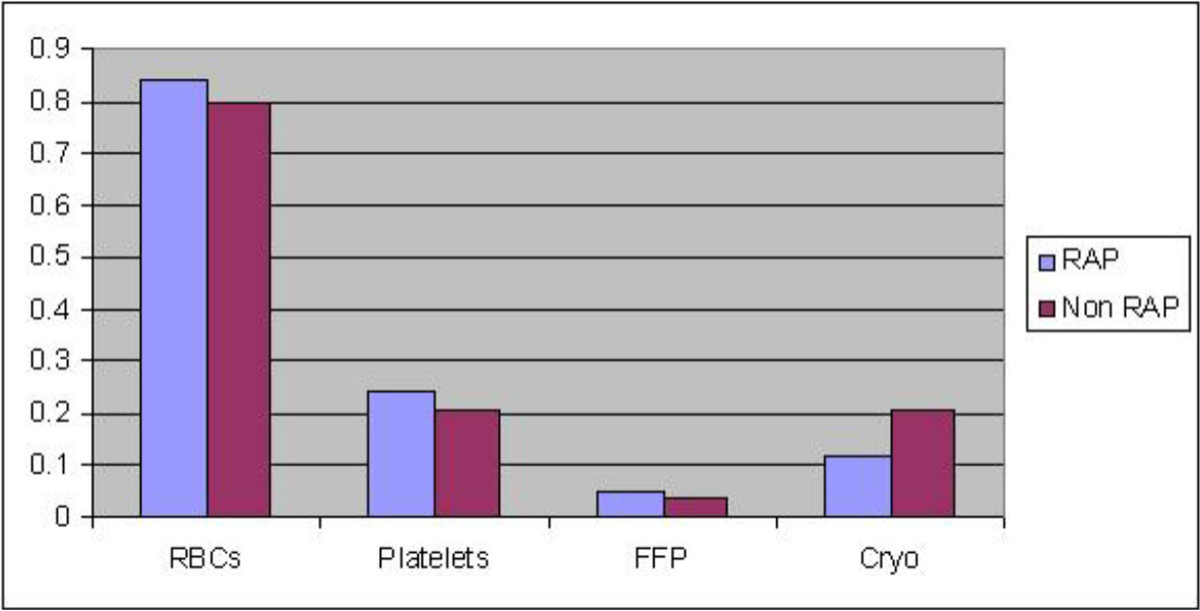


**Figure 3:**